# Analysis of DLA-DQB1 and polymorphisms in CTLA4 in Cocker spaniels affected with immune-mediated haemolytic anaemia

**DOI:** 10.1186/s40575-015-0020-y

**Published:** 2015-06-09

**Authors:** Anna J. Threlfall, Alisdair M. Boag, Francesca Soutter, Barbara Glanemann, Harriet M. Syme, Brian Catchpole

**Affiliations:** Department of Clinical Science and Services, Royal Veterinary College, North Mymms, Hatfield, AL9 7TA Hertfordshire UK; The Royal (Dick) School of Veterinary Studies, The University of Edinburgh, Easter Bush Campus, Midlothian, EH25 9RG UK; Department of Pathology and Pathogen Biology, Royal Veterinary College, North Mymms, Hatfield, AL9 7TA Hertfordshire UK

**Keywords:** Dog Leukocyte Antigen (DLA), Cytotoxic-T-Lymphocyte-Antigen-4 (CTLA4), Cocker spaniel, Immune Mediated Haemolytic Anaemia (IMHA)

## Abstract

**Background:**

Cocker spaniels are predisposed to immune-mediated haemolytic anaemia (IMHA), suggesting that genetic factors influence disease susceptibility. Dog leukocyte antigen (DLA) class II genes encode major histocompatibility complex (MHC) molecules that are involved in antigen presentation to CD4^+^ T cells. Several DLA haplotypes have been associated with autoimmune disease, including IMHA, in dogs, and breed specific differences have been identified. Cytotoxic T lymphocyte antigen 4 (CTLA4) is a critical molecule involved in the regulation of T-cell responses. Single nucleotide polymorphisms (SNPs) in the CTLA4 promoter have been shown to be associated with several autoimmune diseases in humans and more recently with diabetes mellitus and hypoadrenocorticism in dogs. The aim of the present study was to investigate whether DLA-DQB1 alleles or CTLA4 promoter variability are associated with risk of IMHA in Cocker spaniels.

**Results:**

There were a restricted number of DLA-DQB1 alleles identified, with a high prevalence of DLA-DQB1*007:01 in both groups. A high prevalence of DLA-DQB1 homozygosity was identified, although there was no significant difference between IMHA cases and controls. CTLA4 promoter haplotype diversity was limited in Cocker spaniels, with all dogs expressing at least one copy of haplotype 8. There was no significant difference comparing haplotypes in the IMHA affected group versus control group (p = 0.23). Homozygosity for haplotype 8 was common in Cocker spaniels with IMHA (27/29; 93 %) and in controls (52/63; 83 %), with no statistically significant difference in prevalence between the two groups (p = 0.22).

**Conclusions:**

DLA-DQB1 allele and CTLA4 promoter haplotype were not found to be significantly associated with IMHA in Cocker spaniels. Homozygosity for DLA-DQB1*007:01 and the presence of CTLA4 haplotype 8 in Cocker spaniels might increase overall susceptibility to IMHA in this breed, with other genetic and environmental factors involved in disease expression and progression.

## Lay summary

Cocker spaniels are predisposed to developing immune-mediated haemolytic anaemia (IMHA), where red blood cells are destroyed by antibodies produced by the immune system. The breed predisposition suggests genetic susceptibility, and the present study was designed to investigate whether two different immune response genes were involved in increasing the risk of IMHA in this breed. Dog leukocyte antigen (DLA) class II genes code for molecules involved in stimulating the immune system against foreign and potentially host proteins. Several studies have shown that some variants of DLA genes can increase or reduce the risk of developing autoimmune diseases. Cytotoxic T lymphocyte antigen 4 (CTLA4) is a molecule that acts like a ‘braking system’ for the immune system. If this does not work properly, the immune system can become relatively hyperactive. Alterations in the genetic code for this molecule are associated with several diseases in humans and dogs, especially diseases of the immune system. The aim of this study was to investigate whether variation in DLA-DQB1 or CTLA4 gene sequences are associated with risk of IMHA in Cocker spaniels.

Limited variability in DLA-DQB1 alleles was identified in Cocker spaniels and there was no obvious difference comparing dogs that developed IMHA and those that did not. CTLA4 diversity was also limited in Cocker spaniels and there was no significant difference between the IMHA-affected group and the control group.

DLA-DQB1 and CTLA4 variation was limited in Cocker spaniels and was not found to be specifically associated with IMHA. The high prevalence of certain alleles might increase the overall breed susceptibility to IMHA, with other genetic and/or environmental factors acting as a trigger for the disease to develop.

## Background

Immune mediated haemolytic anaemia (IMHA) is a life threatening disease. It is relatively frequently encountered in dogs, with an estimated prevalence of 0.2 % [1]. The disease is characterised by production of anti-erythrocyte antibodies, with or without involvement of complement, and subsequent destruction of red blood cells, either within the circulation (intravascular haemolysis) or in the liver and spleen (extravascular haemolysis). Production of anti-erythrocyte antibodies occurs spontaneously in approximately 65–75 % of cases (primary or idiopathic IMHA) [2–6], or can occur secondary to alterations in erythrocyte antigenicity as a result of a variety of factors (secondary IMHA). Risk factors for secondary IMHA include exposure to drugs/toxins, neoplasia, infections and systemic immune-mediated disorders. There are breed differences in susceptibility to IMHA in dogs, with the Cocker spaniel being overrepresented in several studies [3, 4, 6–10]. The difference in prevalence between breeds suggests that genetic factors might play a role in determining susceptibility to IMHA in dogs.

MHC class II molecules are central to the presentation of antigen for recognition by T lymphocytes: in humans their encoding genes (HLA-D) have been frequently implicated in the pathogenesis of autoimmune diseases [11]. MHC class II variability has been studied extensively in human medicine, and certain human leukocyte antigen (HLA) haplotypes have been found to increase (HLA-DR3 and DR4) or decrease (HLA-DR2) the relative risk for developing specific autoimmune diseases [12–15]. Specifically, HLA-DR3 is associated with Graves’ disease in humans; the frequency of DR3 in Graves’ disease patients was 40–55 % compared to 15–30 % in the general population, resulting in a relative risk for people with HLA-DR3 of 3.4 [12]. More recently, it has been demonstrated that HLA-DR3 is the primary HLA class II allele responsible for susceptibility to type 1 diabetes and autoimmune thyroid disease in families in which both diseases cluster [13]. Similarly, almost all patients with type I diabetes express HLA-DRA 3 and/or HLA-DRA4, and HLA-DR2 is strongly protective; individuals carrying HLA-DR2, even in association with one of the susceptibility alleles, rarely develop diabetes [15]. Some HLA alleles predispose to multiple diseases (HLA-DRA3/4), although there are many HLA allelic associations with individual diseases [14]. The genetic basis of autoimmune haemolytic anaemia is poorly characterised in humans, with limited data available, although there have been conflicting reports of association with HLA-A1, B7 and B8 [16].

Various associations have been identified with the dog leukocyte antigen (DLA) complex and autoimmune disease, including systemic lupus erythematosus (SLE) [17], hypoadrenocorticism [18, 19], canine rheumatoid arthritis [20], hypothyroidism [21–23], diabetes mellitus [24], necrotising meningoencephalitis [25], anal furunculosis [26] and canine IMHA [27]. Specifically the presence of DQB1*007:01 was associated with IMHA and DQB1*020:01 associated with a decreased risk [27].

In humans, genome wide association studies (GWAS) and candidate gene studies have identified associations with polymorphisms in CTLA4 on chromosome 2 and several immune mediated diseases, including type I diabetes, Graves’ disease, Hashimoto’s thyroiditis, hypoadrenocorticism, rheumatoid arthritis and multiple sclerosis [28–32]. Two SNPs in particular have been associated with human type 1 diabetes (+49A/G SNP in exon one and -318C/T SNP found in the promoter region) [33–35].

CTLA4 (CD152) is a cell surface receptor expressed only on primed T lymphocytes and regulatory-T cells. CTLA4 binds CD80/86 ligands, leading to inhibition of T cell activity. CTLA4 is the most important T-cell inhibitory receptor; mice lacking CTLA4 die at a young age due to uncontrolled proliferation of lymphocytes in multiple organs [36].

In dogs, sequencing of the canine CTLA4 gene has revealed a remarkable amount of variation within the 1.5 kbp promoter region, in which 20 SNPs and three insertion/deletions (indels) have been identified, with a total of 17 haplotypes being assigned [37, 38]. In dogs, certain CTLA4 promoter polymorphisms and haplotypes have been associated with both increased and decreased susceptibility to diabetes in multiple breeds [37], and to hypoadrenocorticism in Cocker spaniels [38] and Springer spaniels [39]. Haplotypes 3 and 12 were noted to increase the risk of hypoadrenocorticism in Cocker spaniels (Odds Ratio [OR] = 4.0 and 7.76 respectively) and haplotype 8 was found to decrease the risk (OR = 0.22) [38].

A recent study suggested that HLA and CTLA4 polymorphisms might confer a synergistic risk in the susceptibility to Grave’s disease in humans [40]. A possible link between HLA polymorphisms and expression of CTLA4 has also been suggested in human type 1 diabetes [41], although further studies are required to confirm the association between HLA and CTLA4. Given the likely involvement of CTLA4 in the pathogenesis of canine autoimmune diseases, the variation in DQB1 alleles between Cocker spaniels with and without IMHA [27], and the possible association between HLA and CTLA4 polymorphisms in humans, it was hypothesised that Cocker spaniels with IMHA would be more likely to carry the DQB1*007:01 haplotype when compared to healthy control animals; that polymorphisms in the canine CTLA4 promoter would be implicated in the development of canine IMHA, and that combinations of DLA type and polymorphisms in CTLA4 promoter might increase overall susceptibility to IMHA in Cocker spaniels.

## Results and discussion

### Study population

Forty-six Cocker spaniels affected with IMHA were identified from the Royal Veterinary College (RVC) Canine Genetic Archive. Seventeen dogs were excluded according to our defined criteria. Dogs were excluded due to an equivocal diagnosis, presence of a concurrent infectious condition, neoplasia, recent drug administration, pancytopenia, possible phosphofructokinase (PFK) deficiency or pancreatitis. As a result, 29 Cocker spaniels were included, three of which had evidence of concurrent immune mediated thrombocytopenia (Evan’s Syndrome). Fourteen (48 %) were male (six intact, eight castrated) and 15 (52 %) were female (three intact, 12 neutered). The median age at the time of diagnosis was 7 years (range 9 months to 12 years).

Two hundred and seven Cocker spaniels were identified that were 9 years or older at the time of sample collection and did not have a diagnosis of IMHA. One hundred and forty-one dogs were subsequently excluded based on presence of current or historical immune-mediated or suspected immune-mediated disease and 3 dogs did not have relevant samples available. Sixty-three suitable control dogs were therefore identified from the RVC Canine Genetic Archive. Thirty-one (49 %) of the dogs were male (14 intact, 17 castrated) and 32 (51 %) of the dogs were female (5 intact and 27 neutered). The median age of the control population was 10 years (range 9 to 14 years). The medical conditions for which they presented included neoplasia, intervertebral disc disease, cardiac disease, vestibular disease, epilepsy, cataracts/lens luxation, fracture, chronic kidney disease, pyometra, or polypoid cystitis. Several dogs had more than one concurrent medical condition.

The age distribution of dogs with IMHA was similar to that described in previous studies [4, 7, 10], supporting a predisposition in adult animals. This might reflect the influence of cumulative environmental factors which contribute to the development of IMHA in a genetically susceptible population. A female sex predilection for IMHA in both humans and animals has been postulated [6, 10, 16, 42], and neutered canines may also be predisposed [10, 42]. These findings were not supported in the present study, where a balanced sex distribution was observed in both the IMHA and control populations, and the proportions of neutered animals were similar. It has been suggested that androgens might be protective against autoimmune diseases, and it is possible that the genetic predisposition in Cocker spaniels negates any protective effect provided by androgens.

### Case-control association study of DLA-DQB1 alleles in Cocker spaniels

**Fig. 1:**
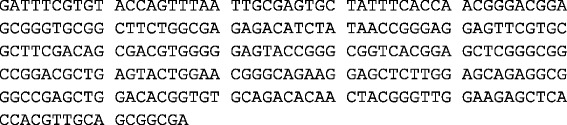
DQB1*lk007cs DNA sequence

Restricted variability of DLA haplotypes was identified in Cocker spaniels [Tables 1 and 2]. The majority of dogs had at least one copy of DQB1*007:01 (27/29 IMHA cases; 93 % and 59/63 controls; 92 %). There was no significant difference when the prevalence of homozygosity for DLA-DQB1 (DQB1*007:01*007:01 or DQB1*020:01*020:01) was compared between the IMHA and control populations (*p* = 0.26). DQB1*020:01 was relatively more prevalent in Cocker spaniels without IMHA (23/63; 37 %), when compared with Cocker spaniels affected with IMHA (6/29; 21 %), although there was no statistically significant difference identified (*p* = 0.15). A new haplotype was identified in this study, which has not been documented previously. This was similar to DQB1*007:01, and has also been recently identified in a population of Cocker spaniels at the Centre for Integrated Genomic Medical Research in Manchester (Kennedy L., personal communication). This DLA haplotype appears specific to Cocker spaniels and has been allocated the temporary name DQB1*lk007cs [Fig. 1]. There was no difference in DQB1*lk007cs frequency between Cocker spaniels with IMHA and the control population.

**Table 1:** DLA-DQB1 allele frequency in Cocker spaniels with (cases) and without (controls) IMHA

DQB1 allele	Cases (2n = 58)	Cases (frequency)	Controls (2n = 126)	Controls (frequency)	*P* value
DQB1*002:01	1	0.017	2	0.016	1.000
DQB1*007:01	41	0.707	81	0.643	0.502
DQB1*lk007cs	2	0.034	5	0.040	1.000
DQB1*012:01	1	0.017	4	0.032	1.000
DQB1*013:02	0	0	1	0.008	1.000
DQB1*013:03	2	0.034	2	0.016	0.600
DQB1*020:01	7	0.121	24	0.190	0.293
DQB1*020:02	1	0.017	3	0.024	1.000
DQB1*021:01	1	0.017	2	0.016	1.000
DQB1*023:01	1	0.017	0	0	0.315
DQB1*08:01:1	1	0.017	2	0.016	1.000

**Table 2:** DLA-DQB1 allele combinations in Cocker spaniels with (cases) and without (controls) IMHA

DQB1* allele combination	Cases (*n* = 29 dogs)	Cases (frequency)	Controls (*n* = 63 dogs)	Controls (frequency)	*P* value
007:01*002:01	1	0.034	1	0.016	0.533
007:01*007:01	14	0.483	23	0.365	0.361
007:01*lk007cs	2	0.069	5	0.080	1.000
007:01*012:01	1	0.034	3	0.048	1.000
007:01*013:03	2	0.069	2	0.032	0.588
007:01*020:01	5	0.172	18	0.286	0.306
007:01*020:02	0	0	2	0.032	1.000
007:01*021:01	1	0.034	2	0.032	1.000
007:01*08:01:1	1	0.034	2	0.032	1.000
012:01*020:01	0	0	2	0.032	1.000
020:01*013:02	0	0	1	0.016	1.000
020:01*020:01	1	0.034	1	0.016	0.533
020:01*020:02	0	0	1	0.016	1.000
020:02*023:01	1	0.034	0	0	0.315
Homozygous	15	0.517	24	0.381	0.260
Heterozygous	14	0.483	39	0.619	0.260

Overall DLA diversity is limited in Cocker spaniels [43]. The DLA-DQB1 locus was studied based on the evidence that there is more variation when compared with the DRB1 and DQA1 loci, and sequencing of DQB1 provided a method of distinguishing between the two most commonly reported haplotypes in Cocker spaniels (DLA-DRB1*006/DQA1*005/DQB*007 and DLA-DRB1*006/DQA1*005/DQB*020). The data presented here were similar to previous findings [27], whereby the DQB1*007:01 allele occurred more frequently in IMHA affected animals when compared to healthy individuals, and the DQB1*020:01 allele was more frequent in the control population, although no statistical information was published on these data [27]. This should be interpreted with caution until larger studies are undertaken.

Homozygosity for MHC-haplotypes has previously been associated with higher risk of autoimmune disease in Italian greyhounds [44], in chronic superficial keratitis [45], in anal furunculosis in German shepherd dogs [26], in SLE related disease complex [17] and hypoadrenocorticism in Nova Scotia duck tolling retrievers, Labradors and West Highland white terriers [18, 19], and necrotising meningoencephalitis (NME) in pugs [25]. The molecular mechanism behind the increased risk associated with homozygosity, independent of haplotype, is not fully understood, although some hypothesise that it is a gene dosing effect, so having two copies of a risk allele or haplotype increases the relative risk further [19]. A recent study used to analyse associations with DLA type in three canine autoimmune diseases utilised a different methodology to target the extended DLA region [46]. An expanded DLA-wide SNP genotyping assay was developed which contradicted previous DLA associations in weimeraners with hypertrophic osteodystrophy (HOD) and Nova Scotia duck tolling retrievers with hypoadrenocorticism, although supported previous DLA associations found in NME in pugs [46]. Further investigation of DLA variation in Cocker spaniels, and other breeds of dogs susceptible to IMHA, might be warranted, utilising this methodology.

### Case-control association study of CTLA4 promoter polymorphisms in Cocker spaniels

Restricted variability in CTLA4 promoter SNPs, haplotypes and genotypes was identified in Cocker spaniels [Tables 3, 4 and 5]. Minor allele frequencies for promoter SNPs were low, with no statistically significant difference seen comparing cases and controls [Table 3]. All dogs had at least one copy of CTLA4 haplotype 8 (Fig. 3). Homozygosity for haplotype 8 was common in Cocker spaniels with IMHA (27/29; 93 %) and in controls (52/63; 81 %), with no statistically significant difference in prevalence between the two groups (*p* = 0.22). CTLA4 promoter polymorphisms were not found to be associated with susceptibility to IMHA in Cocker spaniels in this study. Whilst all dogs had at least one copy of haplotype 8, relatively more heterozygous dogs were identified in the control population, which might suggest that homozygosity for haplotype 8 could increase overall susceptibility to IMHA in this breed. This finding was not statistically significant, which possibly reflects the relatively low power of the study, with a dominant haplotype in this breed. Therefore, a much larger number of dogs might identify CTLA4 promoter polymorphisms that alter susceptibility for IMHA. In contrast, the presence of haplotype 8 reduces the risk of the development of hypoadrenocorticism in Cocker spaniels [38], and a similar trend might have been anticipated for IMHA in the same breed, given the role of CTLA4 as a common susceptibility gene in several autoimmune diseases [47]. Further investigations in other susceptible breeds, such as the English springer spaniel or miniature schnauzer are warranted to better understand whether CTLA4 promoter polymorphisms are implicated in susceptibility to IMHA.

**Table 3:** CTLA4 promoter minor allele frequency in Cocker spaniels with (cases) and without (controls) IMHA

Allele (SNP/INDEL)	Minor allele	Cases minor allele frequency	Controls minor allele frequency	*P* value
SNP 1 (A > G)	A	0.017	0.063	0.276
SNP 2 (A > G)	A	0.017	0.063	0.276
SNP 3 (G > A)	A	0.000	0.008	1.000
SNP 4 (A > G)	A	0.017	0.063	0.276
SNP 5 (A > G)	A	0.017	0.063	0.276
SNP 6 (A > C)	A	0.017	0.063	0.276
SNP 7 (G > A)	G	0.017	0.063	0.276
SNP 8 (G > A)	A	0.000	0.000	1.000
SNP 9 (G > A)	A	0.000	0.016	1.000
SNP 10 (G > A)	G	0.017	0.063	0.276
SNP 11 (C > G)	G	0.000	0.000	1.000
SNP 12 (T > C)	T	0.017	0.063	0.276
SNP 13 (C > T)	T	0.017	0.016	1.000
SNP 14 (C > A)	C	0.017	0.063	0.276
SNP 15 (C > T)	C	0.017	0.063	0.276
SNP 16 (G > T)	G	0.017	0.063	0.276
SNP 17 (T > C)	C	0.000	0.000	1.000
SNP 18 (T > G)	G	0.000	0.000	1.000
SNP 19 (A > G)	A	0.017	0.063	0.276
SNP 20 (C > T)	T	0.000	0.000	1.000
INDEL 1	+	0.000	0.000	1.000
INDEL 2	-	0.017	0.063	0.276
INDEL 3	+	0.034	0.079	0.345

**Table 4:** CTLA4 promoter haplotype frequency in Cocker spaniels with (cases) and without (controls) IMHA

Haplotype	Cases (2n = 58 haplotypes)	Cases (frequency)	Controls (2n = 126 haplotypes)	Controls (frequency)	*P* value
2	0	0.000	2	0.159	1.000
3	1	0.017	6	0.048	0.436
8	56	0.966	115	0.913	0.234
12	1	0.017	2	0.159	1.000
15	0	0.000	1	0.008	1.000

**Table 5:** CTLA4 promoter genotype frequency in Cocker spaniels with (cases) and without (controls) IMHA

Genotype	Cases (*n* = 29 dogs)	Cases (frequency)	Controls (*n* = 63 dogs)	Controls (frequency)	*P* value
8/2	0	0.000	2	0.032	1.000
8/3	1	0.034	6	0.095	0.426
8/8	27	0.931	52	0.825	0.215
8/12	1	0.034	2	0.032	1.000
8/15	0	0.000	1	0.016	1.000
Homozygous	27	0.931	52	0.825	0.215
Heterozygous	2	0.069	11	0.175	0.215

The promoter region of the CTLA4 gene was analysed in this study, based on the evidence that very limited genetic diversity has been identified in the coding region in dogs, and there is a large amount of variability within the promoter region [37]. The population of dogs investigated in the CTLA4 SNP discovery study included 93 dogs of ten breeds [37]. There were seven American Cocker spaniels, but no English or working Cocker spaniels. It is possible that there is variability within the coding sequence that is breed specific and therefore has not yet been identified. Further SNP discovery studies are warranted in a larger pool of dogs to establish the full extent of canine CTLA4 polymorphisms.

### Relationship between DLA-DQB1 alleles and CTLA4 promoter polymorphisms in Cocker spaniels affected with IMHA

Limited variability in DLA-DQB1 alleles and CTLA4 promoter haplotypes was identified in Cocker spaniels in this study. There were considerable numbers of dogs that were homozygous for both DLA-DQB allele and CTLA4 promoter haplotype in both the IMHA (15/29; 52 %) and control populations (22/63; 35 %), with no statistically significant difference between the two groups (*p* = 0.17). The genes encoding DLA-DQB1 are on chromosome 12 whereas the genes encoding CTLA4 are situated on chromosome 37; DLA-DQB1 and CTLA4 are therefore not in linkage disequilibrium.

### Limitations

There were several limitations of this study. Cocker spaniels in both the case and control populations were identified on the basis of owner reporting, rather than Kennel Club registration; which may have introduced a degree of bias, and the possibility of false associations. Kennel Club registration details or pedigrees are rarely available when animals present to a veterinary referral hospital, and the population of Cocker Spaniels herein is considered representative of the population seen in the veterinary practice setting. It should be noted that the relatedness between dogs was not established. The Queen Mother Hospital for Animals is a large referral hospital, accepting cases from a varied population of animals across the south of England and whilst there remains no guarantee that patients were not closely related, this was considered unlikely based on scrutiny of the case details. An analysis of the recorded birth dates of included animals suggested that there were no siblings, although birth dates were recorded by the owner, and therefore this could introduce a source of error. Population stratification is a possibility, but considering the limited number of SNPs assessed and their relatively low minor allele frequencies, there was not enough variability in the dataset to be able to perform robust analysis for stratification.

A control population of Cocker spaniels of 9 years old or greater that did not have a history of immune-mediated disease was selected; with the same limitations regarding breed reporting and patient-relatedness appreciated in this population. Klag (1993) and Reimer (1999) demonstrated that IMHA can occur in dogs between 1 and 13 years, with a median of six years of age, and this was also demonstrated in our case population. It is possible that a proportion of the control population might have developed IMHA, or another autoimmune disease, later in life. It is also possible that selection of an older population of controls, compared with the cases might have introduced bias as a consequence of temporal changes in the genetic makeup of the breed, resulting from a popular sire effect, although the relatively small difference in ages between the two groups and the time over which the samples were accrued make this unlikely to have had a major effect. It is important to note that the control population was selected from a clinical patient population, not healthy animals; background information of the control animals was collected from computer records at the Queen Mother Hospital for Animals (QMHA). This information was focussed on the condition for which the animal was referred, and was less likely to document previous history of an autoimmune disease. The combination of these factors might have increased the proportion of animals in the control population with a susceptible genotype.

IMHA is a heterogeneous syndrome, and there might be differences in the specific susceptibility genes that contribute to the different phenotypes. Human autoimmune haemolytic anaemia is commonly subdivided according to the *in vitro* reactivity of the autoantibodies involved, i.e. whether they cause auto-agglutination at +4 °C or at +37 °C [16]. The in vitro reactivity commonly corresponds with the autoantibody type, with the cold disease typically associated with IgM autoantibodies and intravascular haemolysis, and the warm disease associated with IgG. Although less well characterised in dogs, this has been documented [48], and it is possible that specific susceptibility haplotypes might have been identified if the canine population in this study had been subdivided based on the type of autoantibody involved. This was not possible with the current patient cohort that did not undergo Coombs’ testing. A prospective study, with improved case phenotyping could be informative.

In this study, DLA*DQB1 was investigated alone, without assessment of DRB or DQA. Complete DLA typing would be preferable, although would be unlikely to reveal any clear associations in Cocker spaniels with and without IMHA due to the limited DLA diversity in this breed [43].

## Conclusions

A genetic predisposition for IMHA is suspected, based on breed and familial susceptibility. This study confirmed that the Cocker spaniel breed has limited genetic diversity, demonstrated by the predominance of DLA allele DQB1*007:01 and CTLA4 haplotype 8. This lack of diversity within the breed at these two loci creates difficulty in identifying genetic factors that predispose to autoimmunity using the traditional case: control association study approach. Given that Cocker spaniels are predisposed to a number of autoimmune diseases including IMHA [4], keratoconjunctivitis sicca [49], chronic pancreatitis [50] and hypothyroidism [23, 51], it is possible that the breed as a whole is predisposed to autoimmunity, but that other genes and environmental factors then influence disease progression.

We did not identify any association with IMHA and a specific DLA-DQB1 type or CTLA4 promoter polymorphism in Cocker spaniels. It is likely that a larger number of animals are required to unveil subtle differences in the genetic susceptibility for IMHA within this breed. Continued study of IMHA in dogs presents opportunities for comparative and translational research and might help develop a better understanding of the pathophysiology of this complex disease.

## Methods

### Study population

EDTA blood samples were obtained from the genetic archive of the RVC, University of London. Samples were from Cocker spaniels that had been referred to the QMHA between 1^st^ February 2005 and 29^th^ July 2013, and had been archived following completion of diagnostic testing with informed owner consent for their use in clinical research, and with approval from the institutional Ethics and Welfare Committee. Breeds were identified according to the owner report on admission of the patient to the hospital; Kennel Club registration numbers were not available for review. Cocker spaniels were included in the IMHA group population if they presented with a packed cell volume less than 30 %, and also had evidence of immune-mediated destruction of red blood cells (at least one of the following: spherocytosis, positive in-saline agglutination, positive Coombs’ test, haemoglobinuria or hyperbilirubinaemia). Dogs were excluded if they had any known history of exposure to a primary trigger for IMHA, including any history of neoplasia, infection or recent drug therapy. Dogs were also excluded if there was any indication of an erythrocyte enzyme deficiency, or if they had travelled outside of the United Kingdom (UK), unless they had undergone serological examination for vector-borne diseases. Dogs had a minimum of routine haematological and biochemical analyses, coupled with thoracic and abdominal imaging, to exclude underlying diseases as far as possible.

A breed matched control population was identified by examining clinical records of Cocker spaniels referred to the QMHA during the same time period for investigation not associated with autoimmune disease. Control animals were required to be nine years of age or older at the time of sample collection. Dogs were excluded from the control population if they had any known history of immune-mediated disease.

### DNA extraction and polymerase chain reaction (PCR)

Genomic DNA was extracted from EDTA blood using the GenElute Blood Genomic Extraction Kit (Sigma-Aldrich, UK) according to the manufacturer’s instructions. The DNA was then used in PCR to amplify the DLA-DQB1 exon 2 and the CTLA4 promoter region (1.6 kb upstream of exon 1), in separate reactions. Primers were synthesised by Sigma- Aldrich (CTLA4 promoter sense: 5ʹ-TGCTCCTCTGTGGCTATGTG-3ʹ and CTLA4 promoter antisense: 5ʹ-TGAACACTGCTCCATAAAGC; DQB1/M13F sense: 5ʹ- TGTAAAACGACGGCCAGTCTCACTGGCCCGGCTGTCTC-3ʹ and DQB1 antisense: 5ʹ-CACCTCGCCGCTGCAACGTG-3ʹ). The DLA-DQB1 sense primer was tagged at the 5ʹ end with the M13F target sequence to allow sequencing (Source Bioscience, UK) using the company stock primer (M13F 5ʹ-TGTAAAACGACGGCCAGT-3ʹ). CTLA4 primers were designed by the Immunology and Immunogenetics Laboratory at the Royal Veterinary College. PCR was performed in 25 μL reaction volumes for DLA-DQB1 with 1 μL genomic DNA as template and 2 μL of 20 pmol/μL final concentration DQB1-specific primers, and 50 μL reaction volumes for the CTLA4 promoter with 2 μL genomic DNA as template and 4 μL of 20 pmol/μL total concentration CTLA4 promoter-specific primers. Each reaction contained Hi-Spec additive (DLA-DQB1 5 μL; CTLA4 10 μL), ImmoBuffer (DLA-DQB1 2.5 μL; CTLA4 5 μL), MgCl_2_ (DLA-DQB1 1.25 μL; CTLA4 2.5 μL; 2.5 mM final concentration), deoxynucleotide triphosphates (DLA-DQB1 0.25 μL; CTLA4 0.5 μL; 1 mM final concentration) and Immolase DNA polymerase (DLA-DQB1 0.1 μL [1.25 U]; CTLA4 0.2 μL [2.5 IU]); (all Bioline, UK).

Thermocycling conditions consisted of an initial polymerase activation at 95 °C for 10 min, followed by 35 cycles of 94 °C for 40 s (denaturation), 60 °C for 30 s (annealing) and 72 °C for 1 min for DLA-DQB1 and 2 min for CTLA4 (elongation), with a final extension step of 72 °C for 10 min (G-Storm GS1 Thermal Cycler, Gene Technologies, UK).

The DLA-DQB1 PCR products were purified using the GenElute PCR Clean-up Kit (Sigma-Aldrich, UK). CTLA4 promoter PCR products were electrophoresed on a 2 % agarose/1*TBE gel containing 6 % Safe View Nucleic Acid Stain (NBS Biologicals Ltd., UK) and using a 1 Kb molecular weight ladder (Hyperladder I, Bioline, UK). The gels were visualised under UV light (ImageMaster VDS, Pharmacia Biotech/GE Healthcare, UK). Gel extraction was performed using the GenElute Gel Extraction Kit (Sigma-Aldrich, UK) as per the manufacturer’s instructions.

**Fig. 2:**
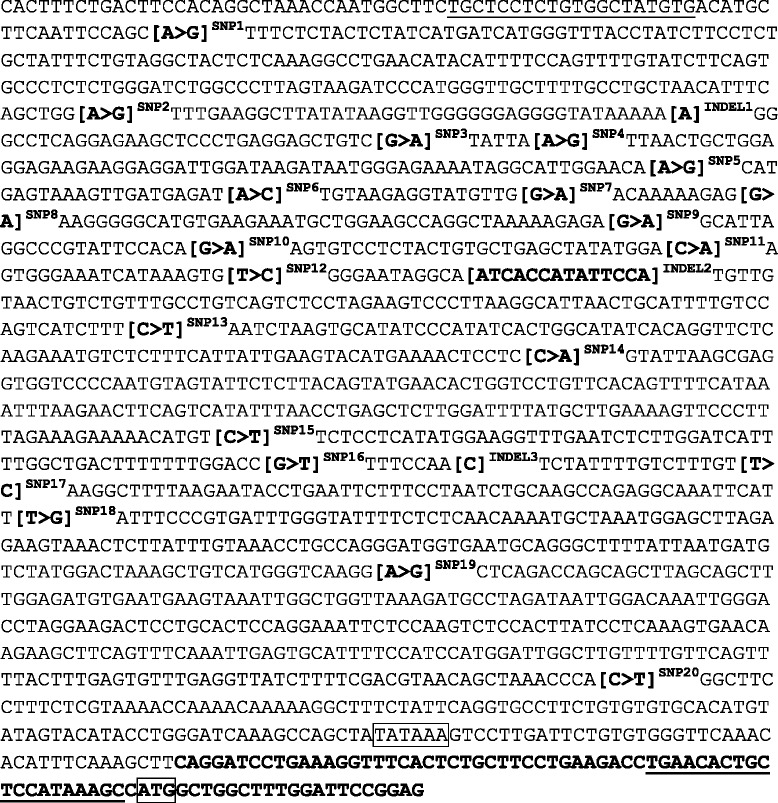
Annotated canine CTLA4 promoter DNA sequence [37, 38]. SNPs and INDELs are labelled and primer-binding sites are underlined. The TATA box and start codon are boxed. The start of the mRNA sequence (NM_001003106.1) is shown in bold text at the 3ʹ end of the sequence. SNPs are shown with the canine genome assembly allele (NC_006619.3) first, followed by the variant allele

**Fig. 3:**
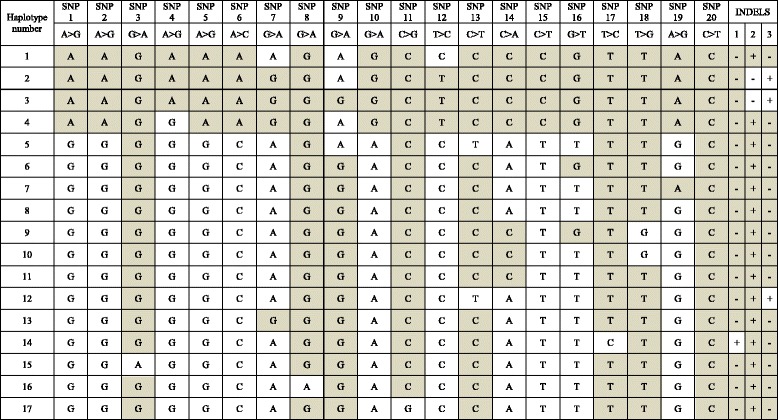
CTLA4 promoter haplotype assignment [37, 38]. SNPs are defined with the canine genome assembly allele (NC_006619.3) first (and shaded within the table), followed by the variant allele

The purified PCR product (DLA-DQB1) and the gel extraction product (CTLA4 promoter) were submitted for sequencing (Source Bioscience, UK). DLA-DQB1 analysis and assignment of alleles was performed using SBT Engine version 2.17 (GenDx). DLA-DQB1 alleles were assigned based on established nomenclature.^1^ CTLA4 promoter chromatograms were analysed using BioEdit Sequence Alignment Editor Software. Previously documented SNPs and indels were analysed and haplotypes assigned for each dog [Figs. 2 and 3].

### Statistical analyses

DLA-DQB1 and CTLA4 promoter allele, haplotype and genotype frequencies were calculated for both groups and compared between cases and controls using the Fisher’s exact test. Associations were considered statistically significant if *p* < 0.05.

### Availability of supporting data

The data sets supporting the results of this article are included within the article (and its additional files).

## Abbreviations

CTLA4: Cytotoxic T lymphocyte antigen 4
DLA: Dog leukocyte antigen
DNA: Deoxyribonucleic acid
EDTA: Ethylenediaminetetraacetic acid
GWAS: Genome wide association study
HLA: Human leukocyte antigen
HOD: Hypertrophic osteodystrophy
IMHA: Immune mediated haemolytic anaemia
INDEL: Insertion/deletion sequence
MHC: Major histocompatibility complex
NME: Necrotising meningoencephalitis
OR: Odds ratio
PCR: Polymerase chain reaction
PFK: Phosphofructokinase
QMHA: Queen Mother Hospital for Animals
RVC: Royal Veterinary College
SLE: Systemic lupus erythematosus
SNP: Single nucleotide polymorphism
TBE: Tris borate EDTA
UK: United Kingdom

## Footnotes

1 See: http://www.ebi.ac.uk/ipd/mhc/dla/.
